# *On your feet*: protocol for a randomized controlled trial to compare the effects of pole walking and regular walking on physical and psychosocial health in older adults

**DOI:** 10.1186/1471-2458-14-375

**Published:** 2014-04-17

**Authors:** Juliette O Fritschi, Wendy J Brown, Jannique GZ van Uffelen

**Affiliations:** 1 School of Human Movement Studies, The University of Queensland, Brisbane, QLD, Australia; 2 Institute of Sport, Exercise and Active Living (ISEAL), Victoria University, Footscray Park Campus, PO Box 14428, VIC 8001 Melbourne, Australia

**Keywords:** Pole walking, Older adults, Health

## Abstract

**Background:**

Physical activity is associated with better physical and mental health in older adults. Pole walking is a form of walking which may have additional health benefits in older adults, because of the addition of hand held poles, and consequent upper limb involvement. However, few studies have examined the potential additional effects of pole walking on physical and psychosocial health in older adults compared with walking. The aim of this study is to compare the effect of a pole walking program with the effects of a walking program, on physical and psychosocial wellbeing, in older adults in assisted living facilities.

**Methods/Design:**

Sixty men and women from assisted living communities over 65 years will be recruited from senior retirement facilities and randomized into a group based, pole walking program, or walking program. The pole walking group will use the Exerstrider method of pole walking. Total duration of the programs is 12 weeks, with three sessions per week, building from 20 minute to 30 minute sessions.

The primary outcome is physical function, as measured by items from the Seniors Fitness Test and hand grip strength. Secondary outcomes include, physical activity levels, sedentary behaviour, joint pain, and quality of life. All outcomes will be assessed before and after the programs, using valid and reliable measures.

**Discussion:**

The study will add to the evidence base for the effects of pole walking, compared with walking, on physical and psychosocial health and physical function, in healthy older adults. This will improve understanding about the feasibility of pole walking programs and its specific benefits in this population.

**Trial registration:**

Australian New Zealand Clinical Trials Registry ACTRN12612001127897.

## Background

Being physically active is associated with better physical and mental health in adults, and it is well documented that there is no age limit to health benefits related to regular physical activity (PA) [1]. Regular PA leads to improvements in cardiorespiratory fitness, muscle strength, endurance and flexibility [2]. It is also associated with a decrease in the overall burden of disease, as well as improvements in psychological wellbeing, quality of life and cognitive functioning [2,3]. In older adults, there is now good evidence that regular PA increases average life expectancy and reduces disability [4,5]. PA which incorporates specific strength, flexibility and balance training, is also associated with a reduction in the risk of falls in this age group [6,7].

Australian PA guidelines for older adults recommend accumulation of at least two and one half hours of moderate intensity PA on most, preferably all, days of the week for health benefits [8]. US guidelines for older adults add that some PA is better than none, and that older adults who participate in any amount of PA will gain health benefits [5,9]. However, PA participation among older adults is low [10-12]. For example, of Australians aged 65–74 years, only one in three met PA guidelines in 2007–8, and the proportion was just over one in five in those over 75 years [13]. The proportion of adults aged over 65 years is expected to increase from 13% of the total Australian population in 2007 to between 23% and 25% in 2056 [10]. Consequently, there will be a significant increase in the number of older adults who could potentially obtain health benefits from regular participation in PA. It is therefore important to find feasible ways for older adults to increase their PA levels.

Walking is one PA option for older adults, as it can be undertaken regardless of age, health status, and ability [14,15]. It is the most frequently reported form of PA in this population group [16,17]. For example, data from the US Behavioural Risk Factor Surveillance System (BRFSS) show that 44% of men, and 45% of women, aged over 65 years, reported leisure time walking in 2000 [18]. In addition, walking is the most frequently reported activity among older adults who meet the US PA guidelines/recommendations [18]. In Australia, walking for leisure is reported by 46% of adults over 65 years, and of those, 53% engage exclusively in walking [17]. Walking at, or above, 3–4 km per hour is categorized as moderate intensity PA [19], and confers health benefits when recommended frequencies and durations are adhered to.

Pole walking (PW) is an outdoor, non-competitive activity. It is a form of walking, with the addition of hand-held poles, which utilizes upper body muscles [20]. It has similar low impact, moderate intensity characteristics to walking [21]. There are several additional effects of PW compared with moderate intensity walking. During PW, the average oxygen uptake, heart rate, and caloric expenditure are higher than for walking at the same speed [21-23]. Importantly, these additional benefits are achieved without significantly increased perceived exertion [22,24-26]. Evidence of a reduction in knee joint loading when PW is ambiguous [27-29], although some studies have shown lower knee joint forces in participants who walk with poles than in those who don’t [30,31]. The use of poles may provide extra stability for walkers and reduce falls or fear of falls. However, to our knowledge, no studies have measured balance and stability during PW. Because of these characteristics, PW appears to be a suitable form of PA for older adult populations.

PW is used in PA programs by community and government organisations in several countries, and many participants in these programs are older adults [32-34]. For example, 44% of older Polish sport and recreation session participants at Universities of the Third Age attended PW sessions [32]. A recent systematic review of the effects of PW on health found a number of randomized controlled trials of the effects of PW in a range of both clinical and non-clinical populations [35]. These include middle aged, non-obese women [36], adults with type 2 diabetes [37,38], cardiovascular disease [24], peripheral artery disease [39,40], musculo-skeletal conditions [41,42], chronic obstructive pulmonary disease [43], Parkinson’s disease [44,45], Sjogren’s syndrome [25] and breast cancer [46]. Most of these intervention studies lasted between 8 and 24 weeks, were of moderate intensity, and conducted 2–3 times per week [35]. This found that PW is simple, feasible, and effective, and has several beneficial physical and psychosocial effects in mid to older aged adults [35].

There are a number of different PW techniques. The Nordic walking technique, which emerged from the sport of cross country skiing, is practiced and taught throughout the world [47]. In the United States, another style of PW, known now as the Exerstrider method, has developed separately from Nordic walking in Europe [48]. The Nordic walking technique uses a longer stride length and greater hip range of motion than regular walking, and a grasp/release hand grip. The Exerstrider method uses a normal gait, a high forward arm position, and a continuous hand grip. There are indications that the Nordic walking technique is more difficult for older people than Exerstriding [45,49]. For example, Figard-Fabre et al. found that, in obese mid-aged women, after four weeks of Nordic walking training, fewer than 50% of the participants were able to grasp three of the eight technical characteristics of the technique [49]. In another study of adults with Parkinson’s disease, many participants had difficulties with the Nordic walking technique [45]. These difficulties may also be experienced by older adults, who have shorter stride length, and smaller hip joint range of motion than younger adults [50].

Although PW seems to be a suitable form of PA for older adults, few studies have examined the effects of PW on physical and psychosocial health in exclusively older adult populations [35]. To our knowledge, only one study has examined the effects of PW in healthy adults aged over 65 years [51]. This study found significant improvements in functional capacity, but not in gait parameters, or walking speed, in older adults who walked twice weekly for nine weeks, compared with a non-exercise control. In addition, few studies have compared the effects of PW with regular walking (RW) in older adults [51,52]. Therefore, the aim of this trial is to compare the effects of PW with the effects of RW, on physical function, physical activity and sitting time, and wellbeing, in adults aged 65 years or over. The null hypothesis is that there is no difference in these outcomes between participants in the PW group and the RW group.

## Methods/Design

### Design

**Figure 1:**
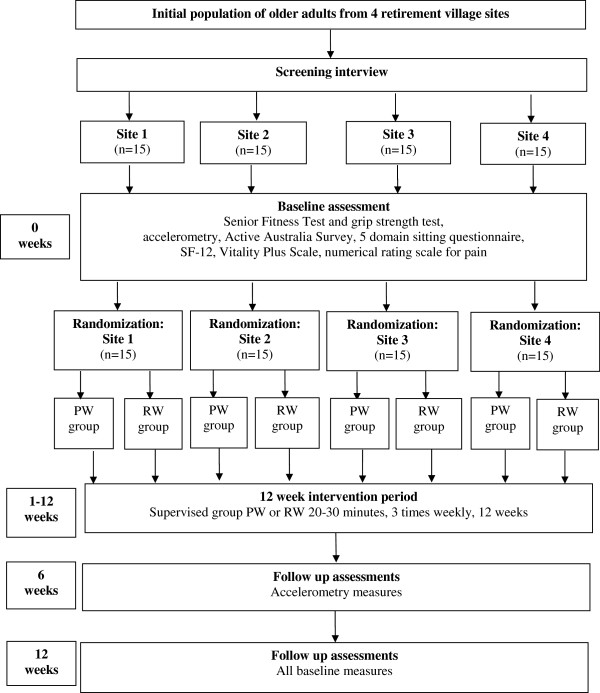
**Overview of study design and timeframe.** (PW = pole walking; RW = regular walking; n = number; SF12 = 12 Item Short-Form Health Survey).

An overview of the study design and timeframe is found in Figure 1. The study is a randomized controlled trial with two arms: a PW program; and a RW program. The study protocol was approved by the Research Ethics Committee at The school of Human Movement Studies, The University of Queensland.

### Study sample and recruitment

Participants will be recruited from four senior living facilities at different locations, but with similar environmental characteristics. The lead researcher will initially contact management staff in the senior living facilities by phone. This phone contact will be followed by a personal visit to the facility managers to introduce the study and the lead researcher. An “Active Aging” presentation will then be offered to the residents of the villages. The presentation will consist of information about the benefits of PA for older adults. The study will be explained in detail and an opportunity for attendees to ask questions and register their interest in participating will be given at the end of the presentation. All attendees will be given an information brochure about the study and the eligibility criteria.

People interested in participating will be contacted personally by the lead researcher. She will then provide any additional information and explanations participants may require, and will screen potential participants for eligibility. Inclusion criteria are: aged 65 years or older. Exclusion criteria include: medically unfit to participate in a walking program; unable to speak or understand English; having a shoulder or elbow flexion range of motion (ROM) of less than 90 degrees; and having pathological conditions of the upper extremity.

In addition to specific verbal or written questions to check the eligibility criteria, the lead investigator will use the Sports Medicine Australia (SMA) pre-exercise screening tool to ascertain medical eligibility to participate in the moderate intensity PA programs [53]. Written informed consent from the participants will be obtained prior to the start of the study.

### Sample size

There are no previous data on the effects of PW compared with RW on physical function and psychosocial health. Sample size estimates were therefore based on the premise that the PW group would achieve changes at least 20% greater than those observed in the RW group, in selected measures of the Seniors Fitness Test (30-second chair-stand test, 30-second arm-curl test, timed up and go test, and a 6-minute walk test) [54]. This difference is thought to be a clinically relevant difference in functional status [52]. Of those subtests, the largest number of participants needed for a stastistically significant 20% difference was for the arm curl test. Based on a 20% difference in normative data for women aged 65–69 years for the arm curl test (mean, 17, SD, 4.1), a power of 0.80 and significance of 0.05, and using the formula n = 2[z*s^2^/Δ^2^], we estimate that 23 participants per group would be needed to detect a between group difference of 20% (i.e. mean, 3, SD, 4.1) in the change score [52].

### Randomization

After baseline assessment of eligible participants at one site, the lead researcher will notify an external researcher of the participant identification numbers. The external researcher will randomly assign 50% of the participants to the PW intervention and 50% to the RW intervention using a random number generator in SPSS and inform the lead researcher of group allocation. This process will be repeated for each site separately. Thus, the total number will be approximately 30 participants in the PW group and 30 in the RW group, with one PW group, and one RW group, with seven to eight participants in each group, at each of four sites.

### Blinding

Outcome measures will be assessed by trained assessors who will be blinded for group allocation before and after the programs. However, participants and exercise instructors will not be blinded because of the difficulty in blinding either of these in trials of specific PA/exercise modalities such as PW [55].

### Outcome measures

Outcome measures will be assessed before commencing the program and at a follow-up testing session one week after the end of the program. The primary outcome measures are selected physical function items of the Seniors Fitness Test (30 second chair stand, 30 second arm curl, timed up and go test, and 6 minute walk test) and grip strength [54]. Secondary outcome measures are behaviour (PA levels and sitting time), and wellbeing (joint pain, quality of life, vitality).

### Primary outcome measures

#### Senior fitness test

The Senior Fitness Test is used to assess physical function, according to standard protocols [54]. This is a widely used test battery for evaluating the effect of exercise interventions in older adults, with 6 subtests which measure the physical abilities needed to perform activities of daily living. However, two of the subtests, for upper and lower limb flexibility, will not be used, as flexibility is not an outcome of interest in this study. Therefore, the tests used in this trial will be: 30-second chair-stand test (the number of times in 30 seconds a participant can stand fully from a seated position without using their arms); 30-second arm-curl test (the number of times a 2.27 kg (5 lb) weight can be curled fully on the dominant side); 2.44 m (8 ft) timed up and go test (the time in which participants can stand from a chair, walk 2.44 m, then return and sit down); and the 6-minute walk test (the maximum distance a participant can in six minutes) [56]. All tests will be measured once, except the timed up and go test, which will consist of a practice, then two trials. The Seniors Fitness Test has acceptable test-retest reliability (R = 0.81-0.98), construct validity against a range of indicators, such as age and exercise status, and criterion validity (r = 0.71-0.82) [54].

#### Hand grip strength test

Hand grip strength is associated with functional limitations, premature mortality, and the development of disability in older adults [57]. Hand grip strength will be measured by the amount of static force that the participant’s dominant hand can squeeze around a dynamometer. A Jamar dynamometer will be used as it is accurate, and shows good inter-rater and test-retest reliability and validity in the older adult population [58,59]. Hand grip strength will be measured in the seated position as per the standard testing protocol approved by the American Society of Hand Therapists (ASHT) [60]. Three trials of grip strength for each hand, with a 60 second rest period between trials, and each with a three second maximum grip, will be conducted and the maximum value recorded [61].

### Secondary outcome measures

#### Behaviour

**Objectively measured PA and sitting time** A tri-axial accelerometer (ActiGraph GT3X+) will be used to assess levels of physical activity and sedentary behaviour in all participants in both the PW and the RW groups before, during (week 6), and at the end of the program (week 12). Participants will be shown by the lead researcher how to position the actigraph accelerometer, which will be worn on an elastic clip-on belt, above the left iliac crest. Participants will be asked to put it on when they first get up in the morning and wear it until going to bed at night. In addition, participants will be asked to complete an activity diary to verify the time that the accelerometer was worn. Valid wear time will be defined as a minimum wear time of 10 hours per day for 4 days [62,63]. Sedentary behaviour will be defined as < 200 cpm, light intensity activity as 200–2689 cpm, moderate intensity activity as 2690–6166 cpm, and vigorous intensity activity as > 6167 cpm [64,65].

##### 

**Self reported PA** The Active Australia Survey is a self-administered survey which is widely used to assess PA in Australian national and state surveys, and intervention studies [66]. Items have acceptable measurement properties for ambulatory older adults [67]. It consists of a set of questions which assess frequency and total time spent walking, and in moderate and vigorous leisure time activity in the past week. Time in each activity is multiplied by a generic metabolic equivalent value of 3.33 METs for walking and moderate activity, and 6.66 METs for vigorous activity, and the sum of all MET.minutes per week is categorized as no PA, (< 33), some PA (33–499), or meeting PA guidelines (≥ 500-999), or high PA (≥ 1000).

##### 

**Self-reported sitting time** Sitting time will be assessed by a five domain sitting questionnaire [68]. The questionnaire assesses the number of hours spent sitting at work, while travelling, watching television, and using a computer when not at work, and during other recreation. These domain specific questions have acceptable reliability and validity [68].

#### Wellbeing

**Pain** Pain levels in the neck, lower back, hip, knee and shoulder joint will be assessed using the numerical rating scale (NRS), consisting of an 11 point interval scale labelled from 0 to 10, with 0 being no pain, and 10 being the worst pain possible [69]. This scale was chosen because it is easy for older adults to understand, and is sensitive to change, valid and reliable [69].

##### 

**Quality of life** The SF12 (12 Item Short-Form Health Survey) is a self-administered questionnaire used to assess quality of life, and it is frequently used as a succinct overall assessment of health [70]. The SF12 has good internal consistency and test–retest reliability in older adults [71]. Two summary scales will be derived, the physical and mental summary scales. They will be scored using norm based methods [70].

##### 

**Vitality** The vitality plus scale is used to assess the perceived benefits of exercise by older adults [72]. It is a self-administered 10 item, multi-dimensional scale, which assesses sleep, energy, aches and pains, restlessness, stiffness, cheerfulness, constipation and appetite. Constructs of vitality relevant to exercise are therefore captured in a concise, reliable, and valid instrument, which is also easy for older adults to use [72].

### Intervention

#### Program duration, frequency and intensity

The exercise sessions will take place at outdoor areas adjacent to the facilities which are convenient to the participants. Program duration is 12 weeks, with a session frequency of 3 times per week. Session durations for the PW and the RW groups will be 20 minutes at the start of the program, increasing to 30 minutes by week 6. Participants will be advised not to change other lifestyle habits, including PA, during participation in the program. The PW and RW sessions will be at different times and/or days so that the groups are separate throughout the program. The exercise sessions will consist of a 5 minute warm up, followed by 20 mins of RW or PW at the first session, and a cool down/stretching period of 5 mins. After six weeks, the RW/PW component will increase to 30 minutes. Participants will be asked to walk at a comfortable intensity. The reason for this is that many of the participants are expected to be frail and non-exercisers. Therefore, to reach a moderate intensity may be unrealistic for them.

#### Pole walking technique to be used

The Exerstrider technique and poles will be used in the PW group. As this PW technique requires a natural gait, continuous hand grip and no arm extension, it has fewer technical requirements for older adults to learn and perform consistently, than the Nordic walking technique [49]. The first exercise session will be used to teach the Exerstrider technique to the PW group, and as an instruction session in the RW group.

#### Group structure and supervision

The intervention programs will consist of supervised group sessions, as there is a positive association between PA maintenance and social support from instructors and group members in older adults [73]. Sessions will be supervised by qualified recreational therapists, who are known to the participants and experienced in leading exercise groups. Both the PW and RW group instructors will receive the same instruction and information concerning the PW and RW session procedures. PW and RW group routes will be the same at each site. In addition, the PW group instructors will be trained in The Exerstrider method. The training package is a standard one developed for use in retirement facilities by the developer of Exerstriding and master trainer of the method (personal communication). Participants in the PW group will receive a free set of Exerstrider poles and training at the beginning of the program. The RW participants will be advised at the beginning of the program that they will be given the opportunity to receive poles and training in their use at the end of the program.

The trial will be monitored by the study leader, who will visit each of the PW and RW groups once weekly to ensure compliance with study protocols. In the case of adverse events, instructors will contact facility medical staff who will arrange for onsite first aid or other intervention as appropriate. The medical staff will inform the study leader within 12 hours. The study leader will register adverse events with the University of Queensland ethics committee within 48 hours.

#### Attendance and dropout

Attendance will be registered at each session by the session supervisor. Participants who do not attend a session will be contacted following the session by the group exercise instructor, and the reasons for their absence will be recorded. If participants indicate that they intend to discontinue the program, the reasons for this will also be recorded, and they will be encouraged to attend the post intervention assessments. If this does not occur, a last measure carried forward protocol will be used.

#### Data analysis

To ensure that randomization resulted in equal distribution of sample characteristics in both intervention groups, baseline characteristics in the intervention and control groups will be compared using t-tests for normally distributed continuous data, appropriate non-parametric tests for non-normally distributed continuous data and chi square tests for categorical variables. Between group differences in study outcomes will be examined using repeated measures of covariance (ANCOVA), adjusted for variables that are associated with both the explanatory and outcome measures; based on previous publications, these may include factors such as age, sex and number of medical conditions.

Both intention to treat analysis, including all participants who were enrolled in the study, and provided both baseline and follow up data, and per protocol analyses, including only participants who completed the program, will be analysed. The level of significance will be set at 0.05. All analyses will be conducted using SPSS version 20 (SPSS Inc, Chicago, IL).

## Discussion

This paper describes the protocol for a randomized controlled trial comparing the effects of PW and RW on physical function, behaviour, pain, wellbeing and social support in older adults. Although effects of PW on fitness have been well-researched [22,23,74,75], no studies have compared the effects of PW with RW on physical function in healthy older adults.

Several different versions of PW exist, and studies have found that different techniques and poles can lead to different outcomes in effectiveness and safety [49]. The choice of the Exerstrider method is a unique feature of this study as it is a simple technique designed for PA, rather than fitness, and thus suited to the older adult population.

In older populations, considerations other than cardiovascular fitness are important for physical and mental health. Maintaining strength to perform activities of daily living, maintain PA levels, and prevent falls, are critical to maintaining independence in older adults [4]. If independence is reduced in this population, quality of life is also reduced and there is an increased risk of institutionalisation [76]. Falls in older adults are often a factor in reduced activity levels, leading to poorer physical function [77]. An activity such as PW, which potentially provides increased stability during exercise compared with RW, may improve overall PA levels and associated health benefits. Thus, PW has the potential to be a safe, effective and easily maintained activity option for older adults. This study will enable better understanding of the potential of PW for increasing PA levels and promoting physical and mental health in healthy older adult populations.

## Abbreviations

PA: Physical activity; PW: Pole walking; RW: Regular walking; ROM: Range of motion; BRFSS: Behavioural risk factor surveillance system; ANCOVA: Repeated measures of covariance.

## Competing interests

The author(s) declare that they have no competing interests.

## Authors’ contributions

JF developed the original design of the study. JF, JvU and WB were involved in further developing the design and the protocol for carrying out the study. JF wrote the first draft of the manuscript. All authors read, edited draft versions and approved the final manuscript.

## Pre-publication history

The pre-publication history for this paper can be accessed here:

http://www.biomedcentral.com/1471-2458/14/375/prepub
